# ROCK inhibitor reduces Myc-induced apoptosis and mediates immortalization of human keratinocytes

**DOI:** 10.18632/oncotarget.11458

**Published:** 2016-08-20

**Authors:** Aleksandra Dakic, Kyle DiVito, Shuang Fang, Frank Suprynowicz, Anirudh Gaur, Xin Li, Nancy Palechor-Ceron, Vera Simic, Sujata Choudhury, Songtao Yu, Cynthia M. Simbulan-Rosenthal, Dean Rosenthal, Richard Schlegel, Xuefeng Liu

**Affiliations:** ^1^ Department of Pathology, Georgetown University Medical School, Washington, DC 20057, USA; ^2^ Center for Cell Reprogramming, Georgetown University Medical School, Washington, DC 20057, USA; ^3^ Department of Molecular and Cell Biology and Biochemistry, Georgetown University Medical School, Washington, DC 20057, USA; ^4^ Department of Biostatistics, Bioinformatics, Georgetown University Medical School, Washington, DC 20057, USA

**Keywords:** Myc, ROCK, cytoskeleton, apoptosis, telomerase

## Abstract

The Myc/Max/Mad network plays a critical role in cell proliferation, differentiation and apoptosis and c-Myc is overexpressed in many cancers, including HPV-positive cervical cancer cell lines. Despite the tolerance of cervical cancer keratinocytes to high Myc expression, we found that the solitary transduction of the Myc gene into primary cervical and foreskin keratinocytes induced rapid cell death. These findings suggested that the anti-apoptotic activity of E7 in cervical cancer cells might be responsible for negating the apoptotic activity of over-expressed Myc. Indeed, our earlier *in vitro* studies demonstrated that Myc and E7 synergize in the immortalization of keratinocytes. Since we previously postulated that E7 and the ROCK inhibitor, Y-27632, were members of the same functional pathway in cell immortalization, we tested whether Y-27632 would inhibit apoptosis induced by the over-expression of Myc. Our findings indicate that Y-27632 rapidly inhibited Myc-induced membrane blebbing and cellular apoptosis and, more generally, functioned as an inhibitor of extrinsic and intrinsic pathways of cell death. Most important, Y-27632 cooperated with Myc to immortalize keratinocytes efficiently, indicating that apoptosis is a major barrier to Myc-induced immortalization of keratinocytes. The anti-apoptotic activity of Y-27632 correlated with a reduction in p53 serine 15 phosphorylation and the consequent reduction in the expression of downstream target genes p21 and DAPK1, two genes involved in the induction of cell death.

## INTRODUCTION

Using standard culture conditions, human foreskin keratinocytes (HFKs) have a limited a life span *in vitro* and serve as a relevant culture system for assaying the molecular events in cellular immortalization. In particular, this model system has been useful for dissecting the immortalization functions of the human papillomavirus (HPV) E6/E7 oncogenes. The E6/E7 oncogenes of the high-risk HPVs are both necessary and sufficient to immortalize HFKs [1–3] and their presence and expression is required for the continued proliferation of HPV-positive cervical cancer cells [4–6]. We have shown previously that Myc can replace a critical function of E6 in cell immortalization (i.e. the induction of hTERT [7, 8]), and similar to E6, Myc cooperates with E7 to efficiently immortalize keratinocytes *in vitro* [9]. While the isolated overexpression of Myc induces apoptosis [10], it is apparent that the anti-apoptotic activity of E7 [11] negates this effect of Myc and thereby permits the continued cell proliferation of Myc/E7 cell cultures [9]. We have published data supporting a model that E7 and the ROCK inhibitor, Y-27632, are components of the same cell pathway involved in cell immortalization [12] and that they share common anti-apoptotic [11, 13–15] and ROCK-targeting activities [14, 16–19].

The Rho/ROCK pathway regulates a plethora of cellular processes including cellular polarity, motility, proliferation and apoptosis [20]. Two mammalian isoforms, ROCK1 (P160ROCK) and ROCK2, have redundant functions, and are differentially expressed in tissues [14]. ROCKs control actin-cytoskeleton assembly and cell contractibility by phosphorylating non-myosin light chain (NMLC) and the actin-binding LIM kinases. As a consequence of such activities, ROCK mediates membrane blebbing, enhances actin-myosin contraction, and activates caspase signaling cascades and cellular apoptosis, including dissociation-induced anoikis. Most recently, we described the use of the Y-27632 ROCK inhibitor and feeder cells to facilitate the long-term propagation of human epithelial cells without perturbing their lineage commitment or differentiation potential [12, 21, 22]. This process has been termed conditional reprogramming [12] and it is highly dependent upon disrupting ROCK functions. To further understand the similarity between E7 and Y-27632 and to understand the role of Y-27632 in facilitating long-term cell proliferation or immortalization, we have explored the possibility that, in addition to generating cell cultures with Myc/E7, we could also generate cell cultures with Myc/Y-27632.

## RESULTS AND DISCUSSION

### Characterization of Myc expression and function in keratinocytes transduced with the myc gene

**Figure 1:**
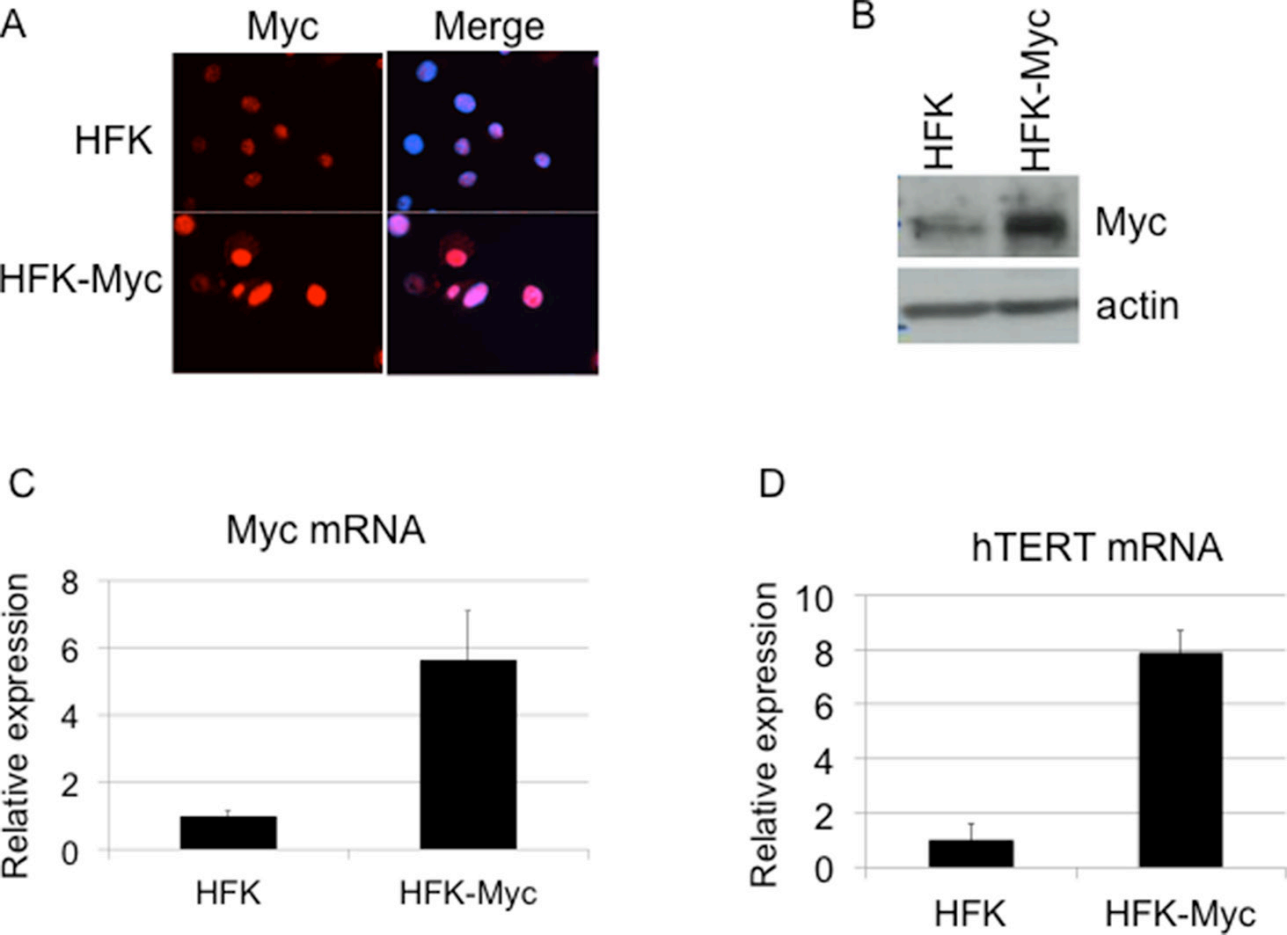
Transduction of Myc and expression of hTERT (**A**, **B**, **C**). Myc expression in Myc transduced keratinocytes. Cells were fixed, permeabilized and expression of the myc gene was detected using anti-Myc antibody by immunofluorescence. Compared to control HFKs, Myc-HFKs expressed increased levels of Myc protein as revealed by immunofluorescent microscopy using the same exposure times, and Myc protein was mainly localized to the cell nucleus (A). Western blot analysis showed increased Myc protein level compare to the control HFKs (B). Total cellular RNA was isolated and myc mRNA level was measured by real time RT PCR using primers specific for myc (C). hTERT expression in Myc transduced keratinocytes (**D**). Total cellular RNA was isolated and hTERT mRNA level was measured by real time RT PCR using primers specific for hTERT. HFK/Myc cells expressed increased level of hTERT mRNA, which confirmed the expression and activity of Myc protein.

Primary human foreskin keratinocytes (HFKs) were stably transduced by a myc retrovirus (HFK/Myc) and after selection the cells were continuously passaged in culture. To confirm expression of Myc protein in HFK/Myc cells, we performed immunofluorescence microscopy and western blot analysis, using an antibody against the Myc protein. The results confirmed that HFK/Myc cells expressed Myc protein primarily in keratinocyte nuclei (Figure 1A) and at elevated levels compared to those of control HFK cells (Figure 1B). Transduced myc gene was detected by quantitative real time PCR (QRT-PCR), which showed increased myc mRNA expression level in HFK/Myc cells (Figure 1C). Since we and other have shown previously that Myc induces hTERT expression and increases telomerase activity [2, 7, 9], we performed QRT-PCR assay to determine expression of hTERT mRNA in HFK/Myc. An eight-fold upregulation of hTERT mRNA was observed in the presence of Myc (Figure 1D), confirming expression and functionality of Myc in keratinocytes.

### Keratinocytes transduced with the myc gene exhibit higher levels of apoptosis and detachment

**Figure 2:**
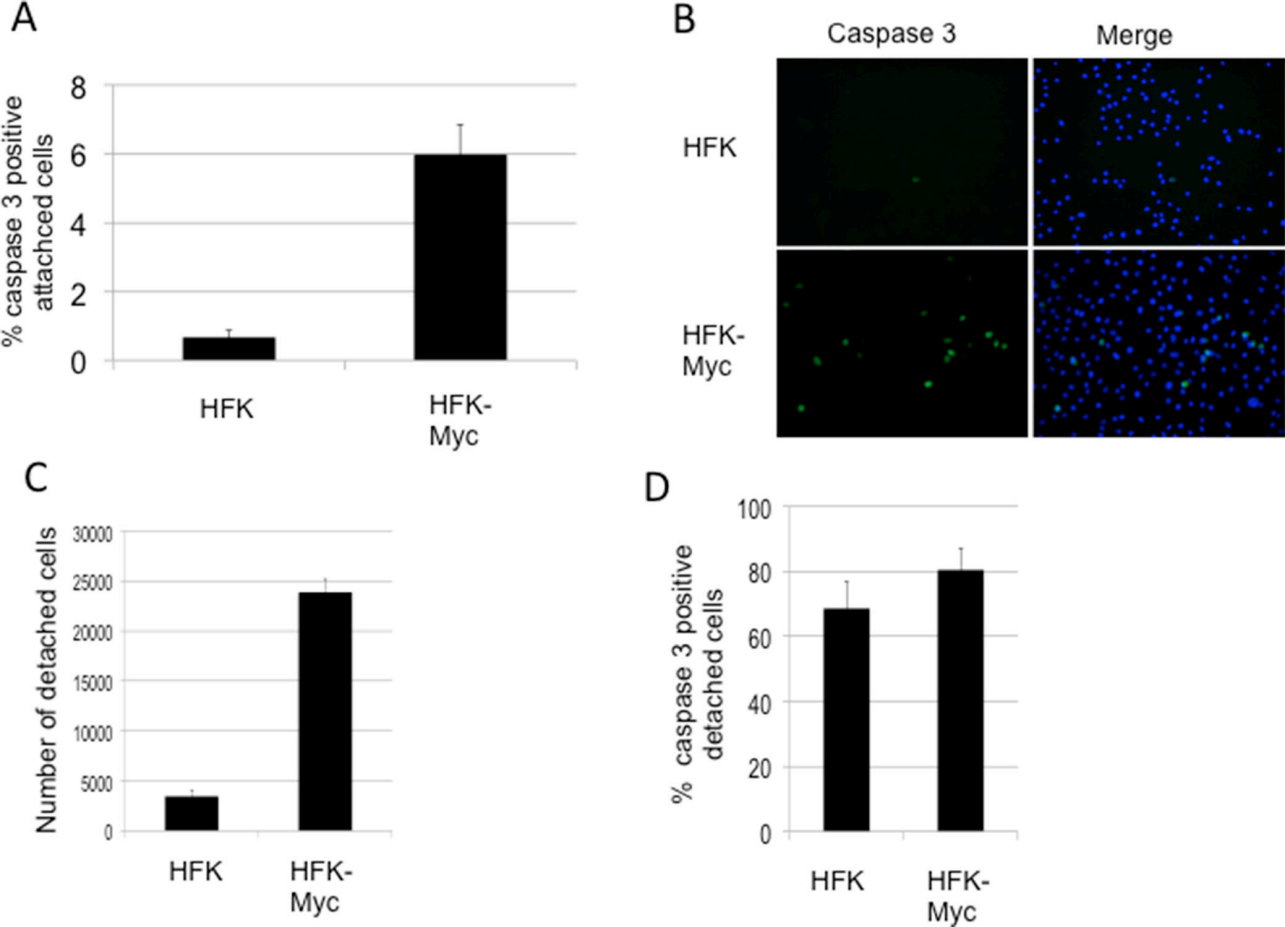
Myc increases apoptosis and detachment of cultured cells (**A** and **B**). Myc-transduced keratinocytes exhibit higher levels of apoptosis as measured by active caspase-3 in attached cells. (**C**) Myc transduced keratinocyte cultures exhibit more detached cells. (**D**). A majority of floaters are apoptotic.

Associated with the high expression levels of Myc, we observed detachment of significant numbers of HFK/Myc cells from the substrate. Myc is well-known to be capable of inducing programmed cell death [10] and in HaCaT keratinocytes, Myc induces apoptosis and sensitizes cells to further apoptotic stimuli [23]. Caspases are important mediators of apoptosis and can be used as a marker for irreversible entry into apoptosis. While different upstream caspases can be activated depending on the apoptotic stimulus, the converging point for almost all apoptotic programs involves the proteolytic activation of procaspase-3 [24]. We therefore measured the level of caspase-3 activity in keratinocytes ectopically expressing Myc using Image-iT Live green caspase detection kit (Life Technologies), according to the manufacturer protocol. HFK/Myc cells showed approximately a 6-fold increase in the number of caspase-3 positive cells that were attached to the substrate in comparison to control HFKs (Figure 2A and 2B). In addition, there are approximately 8-fold more detached cells found in cultures of HFK/Myc compared to those of control HFKs (Figure 2C) and 80% of these cells were apoptotic (Figure 2D). Overall, these data are compatible with HFK/Myc cells undergoing apoptosis while attached to the substrate and subsequently detaching and floating in the medium.

### Y-27632 inhibits apoptosis of Myc-expressing cells

**Figure 3:**
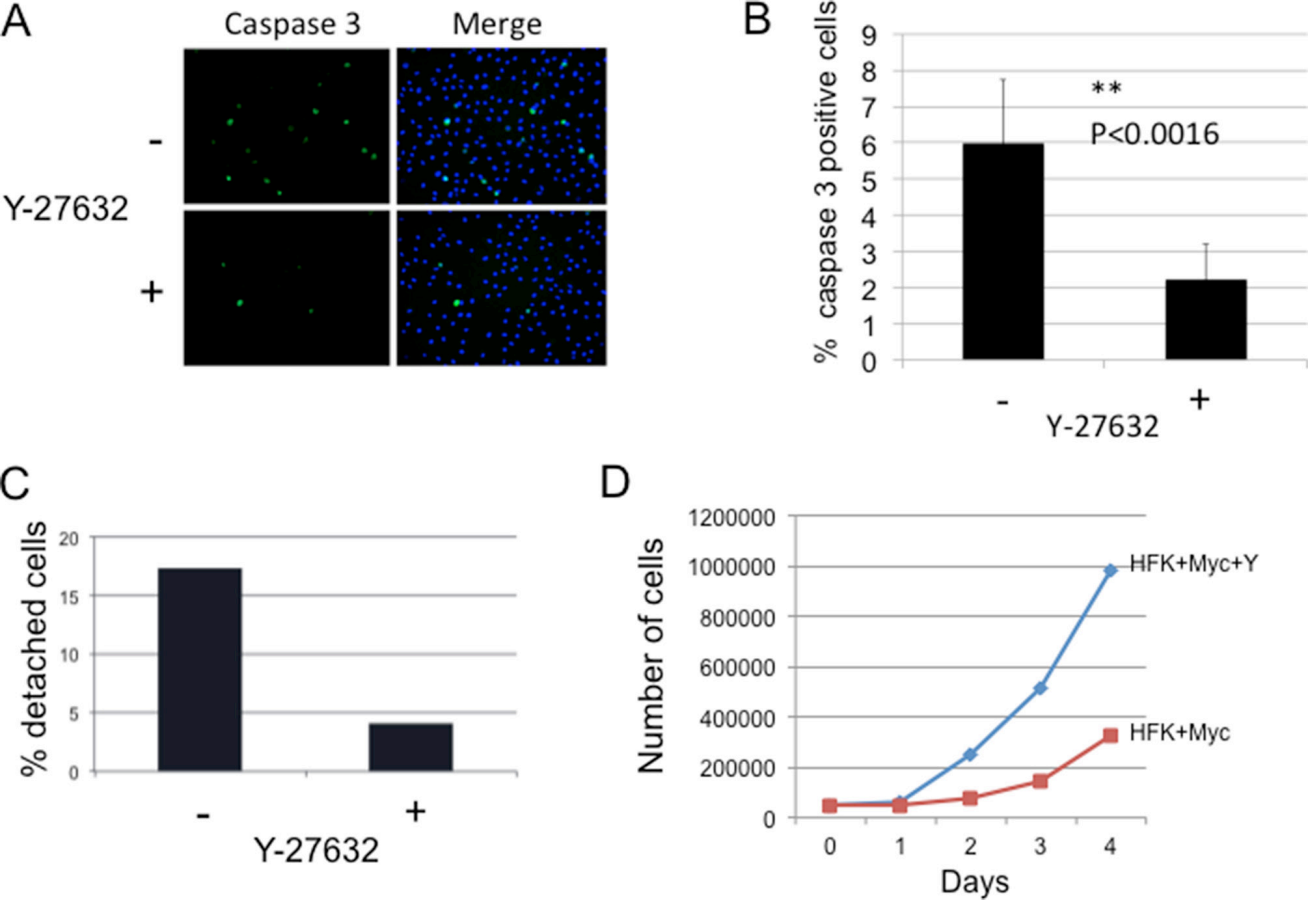
Y-27632 inhibits apoptosis of Myc-expressing cells (**A** and **B**) Y-27632 reduces caspase-3 activity in Myc-expressing HFK (attached cells). The level of caspase-3 activity in attached Myc cells was evaluated by immunofluorescence using Ab against active caspase-3 (Cell Signaling). Cells were stained and positive caspase 3 cells were counted. Apoptosis was significantly inhibited from control in the presence of Y-27632 (*P < 0.0016). (**C**). Y-27632 decreases detachment of Myc-expressing keratinocytes. Myc-HFKs were grown for 3 days in a medium with or without Y-27632. Floating cells and attached cells were collected and counted using a Trypan Blue assay to determine viability. Results showed that Y-27632-treatment significantly decreased the number of detached cells compared to that of the control non-treated Myc-HFKs; while the number of viable attached HFK-Myc cells was increased (**D**).

Myc is known to sensitize keratinocytes to apoptosis [10]. To determine whether inhibition of ROCK by Y-27632 treatment is able to suppress apoptosis in Myc-expressing keratinocytes, cells were treated with Y-27632 inhibitor for 3 days, and attached and detached cells were fixed and immunostained for active caspase-3. Our results show that the caspase-3 activity was much lower in attached cells treated with Y-27632 compared to control untreated Myc cells (Figure 3A and 3B). As shown in Figure 3B, Y-27632-treated cells displayed a 2.69 fold (*p* < 0.0016) decrease in attached caspase-3-positive cells compared to untreated Myc cells. This result implicates Y-27632 as an anti-apoptotic factor in Myc-expressing cells and is in agreement with previous reports of the role of Y-27632 in preventing dissociation-induced apoptosis in hESCs, and increasing cell viability and proliferation [13, 15, 25]. Previous data also indicate that the actomyosin cytoskeleton may be required for caspase-3 activation [17, 26–29], which additionally suggests that inhibition of apoptosis by Y-27632 could be mediated by cytoskeletal rearrangement.

Considering that detachment of Myc-expressing keratinocytes was due to apoptosis described above, we hypothesized that Y-27632 can decrease detached cells. We plated same number of Myc-HFKs in a medium with or without Y-27632 for 3 days, then collected and counted both floating cells and attached cells using a Trypan Blue assay to determine viability. The results showed that Y-27632-treatment significantly decreased the number of detached cells compared to that of the control non-treated Myc-HFKs (Figure 3C), while the number of viable attached HFK-Myc cells was increased (Figure 3D). This increase in culture cell numbers was due to the inhibition of apoptosis and detachment. Increased proliferation did not contribute to this large increase in cell number.

### Y-27632 rapidly reduces membrane blebbing in Myc-expressing keratinocytes

**Figure 4:**
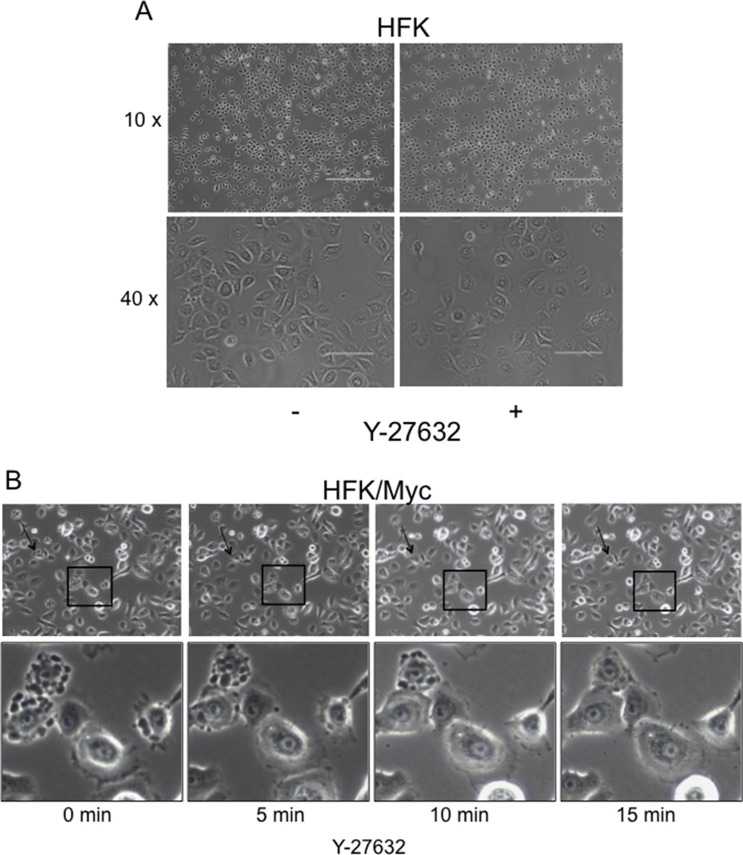
Y-27632 rapidly reduces membrane blebbing in Myc-expressing keratinocytes Plain keratinocytes (**A**) and Myc-expressing cells exhibited a different morphology. Time-lapse images of a representative field of Myc cells at different time periods after treatment with Y-27632 (**B**). Very interestingly, within 5 min after adding Y-27632 membrane blebbing ceased (B). Due to the dynamic process of blebbing and suppression, all cells do not show blebs inhibition at one time. A representative field is selected to include several intensively blebbing cells which are committed to death and some blebbing cells which undergo reversible bleb suppression (panel B, lower). Similar results are seen when keratinocytes were treated with 5 μM blebbistatin (data not shown).

Compared to HFK cells (Figure 4A), HFK/Myc cells exhibit a very specific morphological phenotype when grown in serum-free, defined keratinocyte growth medium (KGM; Figure 4B, left panel). The Myc-expressing cells form blebs on their cell membranes, and these “blebbing” cells gradually increase in number a time- and cell passage-dependent manner (Supplementary Figure S1 and S2). It has been previously reported that F-actin polymerization and actomyosin contractility are necessary for membrane blebbing during the execution phase of apoptosis [16, 30]. In the early stages of apoptosis, caspase-3 cleaves the C-terminus of ROCK which contains the auto inhibitory pleckstrin homology domain (PHD) and Rho-binding domain, rendering it constitutively active to induce MLC phosphorylation and actomyosin contractility, thereby inducing apoptotic blebbing [16, 30, 31]. We therefore evaluated whether Y-27632 could prevent Myc-induced membrane blebbing. We performed live-cell imaging at early time points after addition of Y-27632 to the KGM medium (*i.e*. 5 minutes intervals in the first 15 minutes after Y-27632 application). Our results showed that dynamic membrane blebbing was present in untreated Myc-HFKs (Figure 4, first left panel, Supplementary Figure S2). Very importantly, the addition of the ROCK inhibitor Y-27632 dramatically and rapidly inhibited the membrane blebbing in Myc-HFKs. The suppression of blebbing appears within 5 minutes and is virtually complete after 15 minutes (Figure 4B, last right panel). Thus, while blebbing is an early indicator of apoptosis, it is apparent that the administration of Y-27632 can reverse these changes and restore cells to a normal morphology.

### Rock inhibition decreases stress fibers and phosphorylation of MLC II and cofilin in keratinocytes

To validate that Y-27632 was affecting the cytoskeleton of the keratinocytes, we examined the effect of Y-27632 on the abundance and distribution of filamentous actin (F-actin) stress fibers in HFK/Myc using AlexaFluor 488 phalloidin that specifically binds F-actin. In HFK/Myc cells, F-actin stress filaments were assembled into large radial and circumferential bundles (Supplementary Figure S1A). After treatment with 5 μM Y-27632, the F-actin staining pattern was altered within minutes, resulting in phalloidin staining that was punctuate in appearance, with residual actin filaments greatly rearranged and associated with the cell periphery. An analogous result was obtained with control HFK cells and these changes in F-actin arrangement correlated with cell rounding [20, 32, 33].

To determine the effect of Y-27632 on the ROCK pathway in our HFK/Myc cells, we collected protein samples from HFK/Myc with or without Y-27632 treatment and examined the phosphorylation of downstream targets MLC2 and cofilin by immunoblot analysis. As expected, the phosphorylation of both MLC2 and cofilin level were decreased in cells treated with Y-27632 (Supplementary Figure S3B). These were accompanied by the reduced formation of stress actin as demonstrated previously by immunofluorescent staining of phalloidin (Supplementary Figure S3A).

### Y-27632 suppresses caspase-3 in response to inducers of extrinsic or intrinsic apoptosis

**Figure 5:**
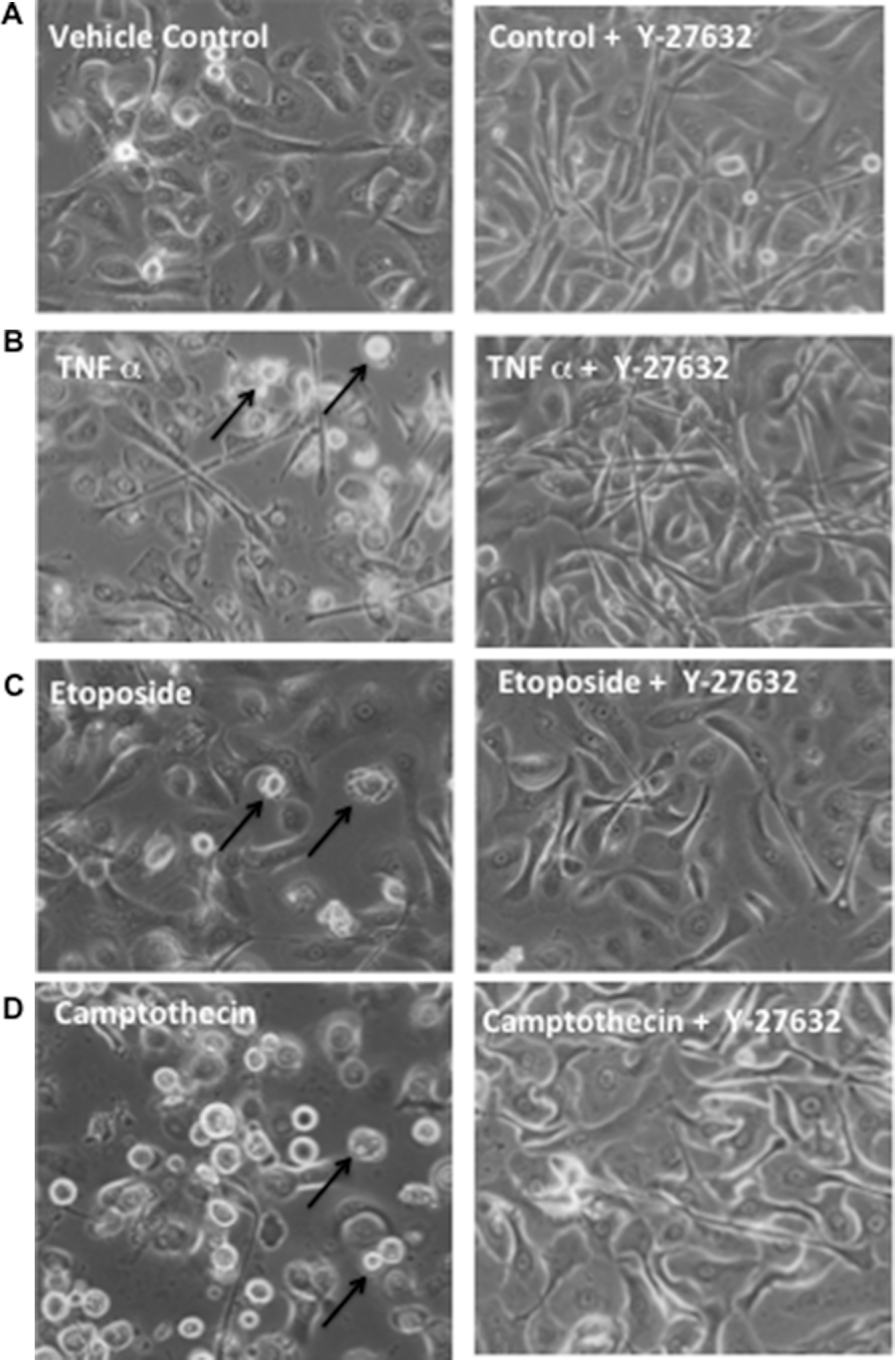
Y-27632 suppresses apoptotic rounding and detachment in response to inducers of extrinsic death receptor or intrinsic mitochondrial apoptosis Primary keratinocytes were established and unexposed (left column) or exposed (right column) to Y-27632 for 24 h. Cells were then left untreated as control (**A**), or treated with α-Fas agonist Ab (**B**), etoposide (**C**), or camptothecin (**D**) for an additional 24 h. Cells were visualized by phase-contrast microscopy for cell morphology and attachment.

**Figure 6:**
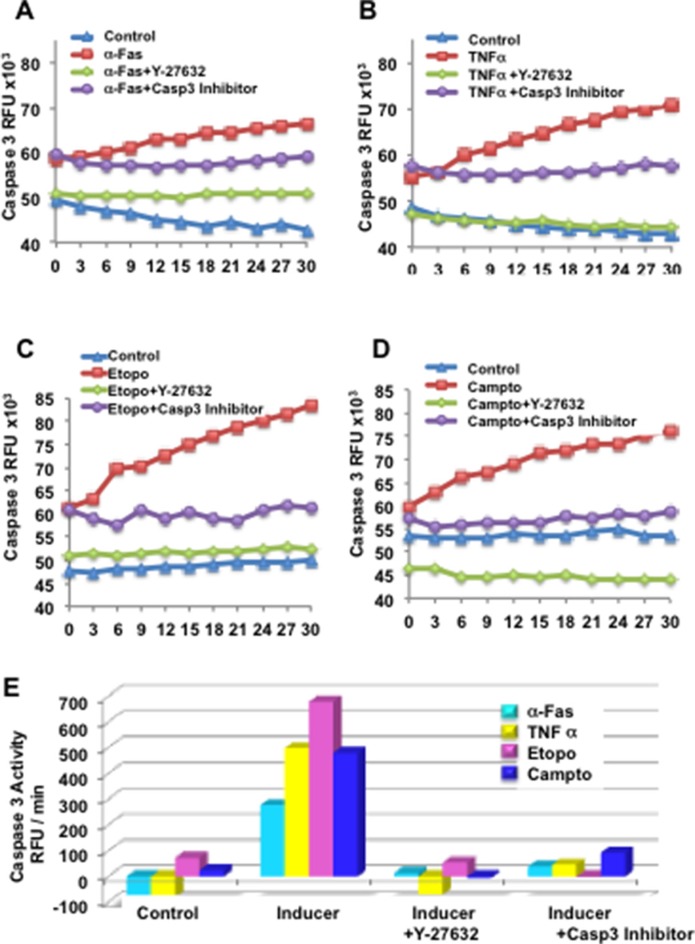
Y-27632 suppresses caspase-3 in response to inducers of extrinsic or intrinsic apoptosis Primary keratinocytes were established and unexposed or exposed to Y-27632 for 24 h, then treated with α-Fas agonist Ab (**A**), TNFα (**B**), etoposide (**C**), or camptothecin (**D**) for an additional 24 h. Cytosolic extracts were derived and subjected to fluorometric caspase-3 activity assays using fluorescent tetrapeptide substrate specific for caspases-3 as described in Materials and Methods. Free AMC, generated as a result of cleavage of the aspartate-AMC bond, was monitored over 30 min. Emission at 460nm from each sample was plotted against time, and linear regression analysis was used to determine the initial velocity (slope) for each curve, which yielded the activity (**E**). Inhibitor of caspase-3 (Z-DEVD-CHO) (BioMol, Plymouth Meeting, PA) was added to cell extracts at a concentration of 50 μM, 10 min prior to addition of the Ac-DEVD-AMC as a control.

Lastly, we queried which apoptotic pathway was inhibited by Y-27632. Apoptosis or programmed cell death can be triggered by two major pathways within the cells: the extrinsic pathway (death receptor pathway) and the intrinsic pathway (mitochondrial pathway) [24]. The extrinsic pathway is activated by ligand-activated death receptors that are members of the tumor necrosis factor (TNF) receptor gene superfamily [34, 35]. Some of ligands and corresponding death receptors include FasL/FasR and TNF-α /TNFR1. The binding of ligand to the receptor activates caspases, cysteine proteases that recognize aspartate at their substrate cleavage site and induce apoptosis [24]. Many anticancer drugs induce apoptosis by molecular mechanisms mediated through mitochondrial dysfunction [36]. Release of cytochrome c from the internal part of the mitochondrial membrane into the cytosol results in the activation of caspase cascades, in particular caspase−9, −3, −6, and −7. Therefore to address this question, we treated HFK with several inducers of the extrinsic apotpotic signaling pathway, including anti-human Fas (250 ng/ml) and tumor necrosis factor - TNF-α (10 ng/ml), as well as common and potent inducers of the intrinsic or mitochondrial apoptotic pathway, such as etoposide (50 μM) and camptothecin (10 μM). Cells were treated with or without 24-h prior exposure to Y-27632. Because caspase-3 is the main executioner of apoptosis, we used the active form of caspase-3 to measure apoptosis. Cytosolic extracts were derived and subjected to caspase-3 activity assays using fluorescent tetrapeptide substrate specific for caspases-3 as previously described [11]. Our results demonstrate that Y-27632 inhibited caspase-3 activity mediated by inducers of both extrinsic and intrinsic apoptosis (Figures 5 and 6).

### Y-27632 results in an “apparent” increase in culture growth rate

Corresponding to the inhibition in apoptosis observed in cultures treated with Y-27632, we also noted an increase in cell accumulation in the cultures with a resultant “apparent” decrease in culture doubling time. We plated 5,000 cells and performed a short, 4 day proliferation assay where we measured the number of attached cells grown under two different conditions (+/−Y-27632). As anticipated, we observed significant increases in the number of attached Y-27632-treated cells compared to non-treated HFK/Myc cells. By day 3 there were 4-fold more attached cells in the treated group (Figure 3D). However, as noted above, this increase in culture cell numbers was due to the inhibition of apoptosis. Increased proliferation did not contribute to this large increase in cell number, as the cell cycle was unaltered (data not shown).

### Y-27632 cooperates with Myc to immortalize human keratinocytes

**Figure 7:**
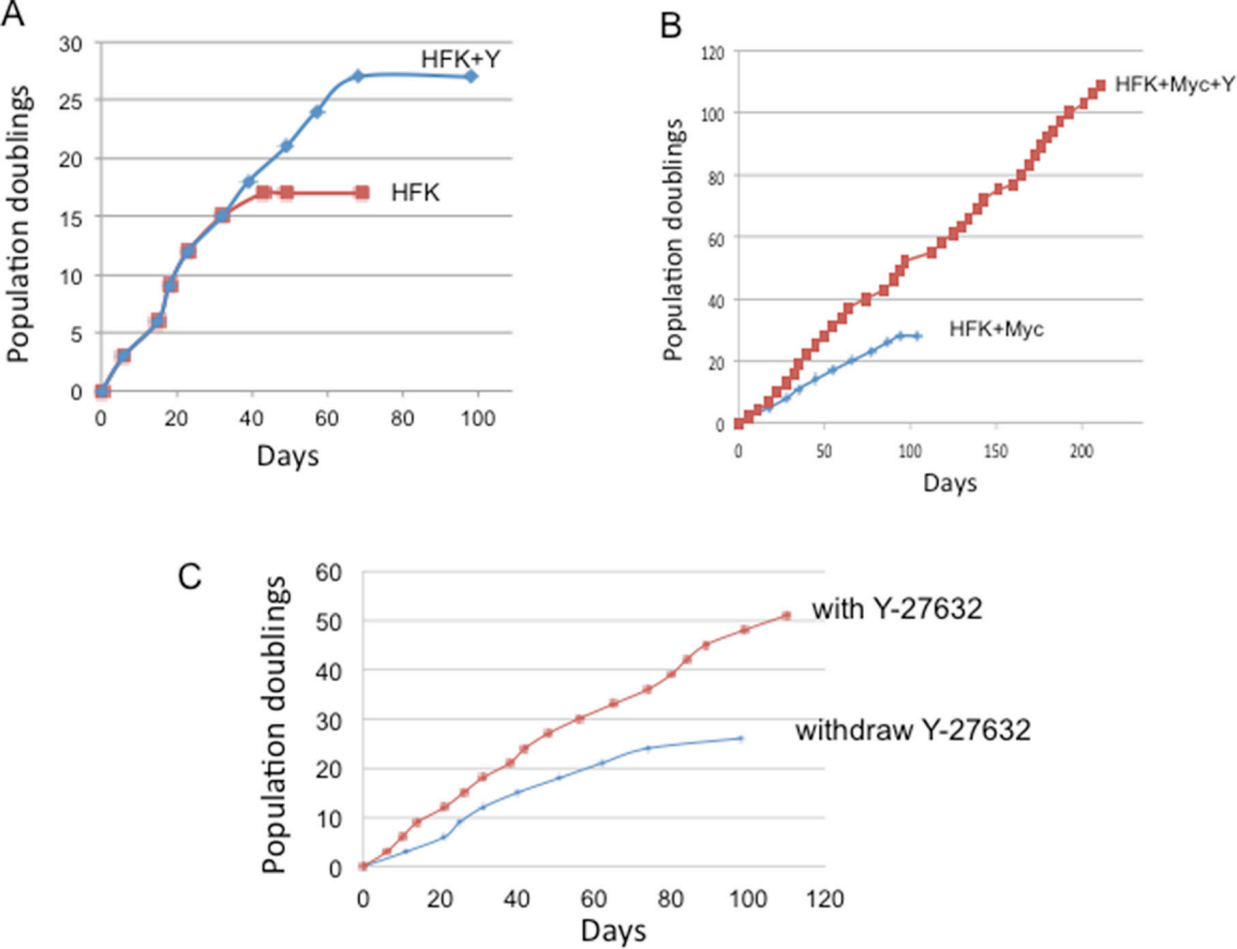
Y-27632 immortalizes Myc-expressing keratinocytes (**A**) Y-27632 is not able to immortalize primary human keratinocytes in synthetic medium (KGM). Primary human keratinocytes were passaged in synthetic medium (Keratinocyte-growth medium) with or without 5 μM Y-27632. (**B**) Y-27632 immortalizes Myc-expressing human keratinocytes. We transduced HFKs with a Myc retroviral vector and after antibiotic selection, cells were passed serially in culture with or without Y-27632 to assay for immortalization. When cells reached ∼80% confluence, they were split at a 1:8 ratio. Therefore, one split corresponds to three cell population doublings. Cumulative population doublings were plotted against time (days). Whereas Myc expressing cells grown in KGM underwent senescence after ∼25–30 PDs, cells grown in synthetic media with Y-27632 have continuously grown for more then 120 PD. These results were confirmed in other 2 independent experiments and new cell lines. (**C**). Withdrawal of Y-27632 decreases growth of Myc-expressing keratinocytes. Y-27632 was withdrawn from the KGM media at the 24 population doublings. Cells continue to grow for 26 additional PDs, and they ultimately enter into senescence.

While Y-27632 significantly promotes the growth of Myc cells treated with Y-27632 via reduction of apoptosis, it was unclear whether these proliferating cells would bypass the Hayflick senescence limit and become immortal. To test this possibility, HFK/Myc cells were cultured with or without addition of Y-27632 in synthetic media (KGM) and were serially passaged. As we expected, control HFK cells became senescent at 17 population doublings. The addition of Y-27632 to these normal HFKs was able to extend cell life span for ∼10 PDs until they underwent senescence at 27 population doublings (Figure 7A). We and others have shown that Myc is unable to immortalize keratinocytes alone [9] and these cells ceased dividing at approximately 25 to 30 population doublings (Figure 7B). However, Myc expression in combination with Y-27632 treatment allowed the keratinocytes to bypass senescence and proliferate long-term (Figure 7B). These experiments were repeated three times with separate preparations of HFKs. These results were also confirmed with Myc mutants (Myc^T58A^, Myc^S62A^ and Myc^T58AS62A^) (Data not shown). Exposure to Y-27632 appears to be required for continued cell proliferation since removal of Y-27632 at 24 population doublings (PDs) eventuates in cell senescence after an additional 25 PDs (Figure 7C).

### Levels of pRB, p16 and hTERT remain unchanged in Y-27632-induced immortalization of HFK/Myc cells

**Figure 8:**
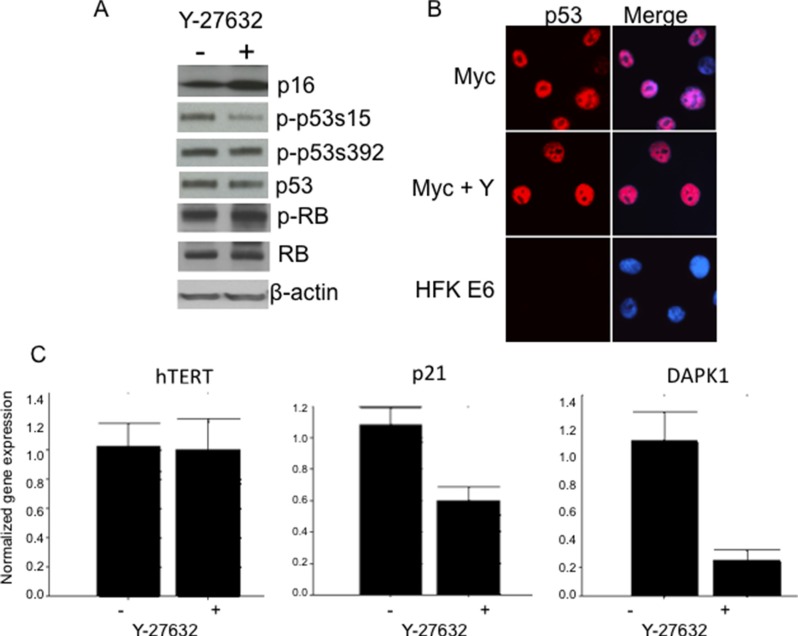
Y-27632 alters phosphorylation of p53 and expression of p53 target genes Y-27632-treated cells retain p53, p16 and phospho-RB protein levels as assessed by immunoblot analysis. Relative protein levels in cells were determined by immunoblot analysis, which showed that immortalized HFK/Myc express similar levels of p16, p53 and phospho-and total pRB protein (**A**). Y-27632 did not alter level and localization of p53 (**B**), HPV E6 expressing was used as control for p53 degradation. Interestingly, p21, which is p53 target protein, was decreased (**C**), which suggest that there is some p53 and pRB independent mechanism of cell cycle regulation. The following antibodies were used to detect the corresponding proteins: p53 (Cell Signal; 1:1,000), pRB (Cell Signal; 1:1,000), and P-pRB (Cell Sign 1:1,000), p16 (Santa Cruz, 1:1,000) and p21 (Santa Cruz, 1:1,000) Secondary antibodies were goat anti-rabbit antibody conjugated to alkaline phosphatase for p53 and pRB or goat anti-mouse antibody for p21, and beta-actin. Beta actin was used as a loading control (Sigma; 1:10,000).

Increased expression of p16^Ink4a^ induces senescence in keratinocytes [37] and conversely, p16 expression is lost *via* deletion or methylation in cancer and transformed cells [38]. Since p16 inactivation was also reported as an important event in keratinocyte immortalization [39–41], we decided to test the status of p16 in immortalized Myc/Y-27632 cells. Interestingly, we detected high levels of p16 in cells in the presence or absence of Y-27632 by immunoblot analysis (Figure 8A). We also examined the level of p-RB and total RB, and similar to p16, there was no significant change in the cells treated with Y-27632 when compared to the parental Myc cells (Figure 8A). These data indicate that Y-27632 does not have a demonstrable effect on critical components of the p16/pRB pathway, which are usually altered during cell immortalization. It cannot be excluded that Y-27632 alters other components of this regulatory pathway. We also examined whether Y-27632 increased telomerase expression, thereby facilitating immortalization. However, QRT-PCR demonstrated that hTERT mRNA levels were unaffected by Y-27632 treatment (Figure 8C, left panel).

### p53 phosphorylated at serine 15, but not total p53 protein, decreases in Y-27632-induced immortalization of HFK/Myc cells

As we determined previously, Myc increases p53 protein levels and thereby sensitizes cells to undergo apoptosis [42]. Since we and others reported that p53 inactivation (degradation) was not critical for HPV-mediated keratinocyte immortalization [1–3, 9], we asked whether abrogation of this pathway was involved in immortalization of Myc expressing cells grown in medium with Y-27632. Unlike HPV-immortalized cells, the immunoblot analysis showed equivalent p53 protein levels in control and Y-27632-treated Myc expressing cells, which supports the hypothesis that p53 degradation is not critical for keratinocyte immortalization (Figure 8A and 8B). This result is similar to our study with Myc/E7 immortalization system [9], this may be due to induction of p53 by Myc and stabilization and inactivation of p53 by HPV E7. Although we did not notice a difference in the level of p53 phosphorylated at serine 392, we did detect a decreased level of phosphorylated p53 at serine 15 (Figure 8A), a residue that is critical for mediating the ability of p53 to transcriptionally regulate its target genes. Thus, our results indicate that Y-27632 apparently inhibits the transcriptional activity and apoptotic signaling of p53 by altering its state of phosphorylation, thereby facilitating cell immortalization.

### Y-27632 decreases expression of p53 targets: p21 and DAPK1

Several p53 targets have been identified for their important pro-apoptotic role [37, 43–48]. One of them is death associated protein kinase gene, DAPK1, which participates in a positive feedback loop activating p53 dependent apoptotic activation [43]. By quantitative real time PCR, we observed a significant decrease in DAPK1 (Figure 8C) upon the Y-27632 treatment of HFK/Myc cells. Interestingly, Raveh et al. [44] reported that p53 mediated apoptotic response induced by Myc was attenuated by DAPK1 inactivation, which supports our observation. Our QRT-PCR data also revealed that the expression of p21, another pro-apoptotic p53 target gene, was significantly decreased after Y-27632 treatment (Figure 8C). These changes strongly suggest that Y-27632 inhibits the transcriptional activity and apoptotic signaling of p53 by reducing p53 phosphorylation at serine 15. Together, our data suggest that, similar to E7 [42], Y-27632 treatment results in a decreased level of proapoptotic genes, which is allows Myc to immortalize cells independent of other cellular or viral oncogenes.

### Y-27632 alters the profile of gene expression associated with apoptosis

In this study, our data demonstrated that Y-27632 reduced Myc-induced apoptosis of human keratinocytes and immortalized Myc-expressing keratinocytes in serum free synthetic medium. To evaluate the spectrum of genes that were altered in expression following exposure to Y-27632, we performed microarray assays and analyzed the categories of genes using DAVID software. The data demonstrated that Y-27632 altered the expression of genes involved in metabolic processes, cellular processes, biological regulation, response to stimuli, cellular component organization or biogenesis, and apoptotic process, as shown in Supplementary Figure S4. Majority of these functional changes are due to gene expression altered by actin dynamics induced by Y-27632. These transcriptional changes can be regulated either by modulating nuclear actin polymerization properties or by impinging on actin-responsive transcription factors. In Supplementary Figure S5, we showed that the IAP antiapoptotic family member survivin was the only apoptosis-related gene which was upregulated at 2, 4, 8 and 24 hrs, while the other 12 genes were downregulated. However, increased p21 and DAPK1 were not observed until 8 hrs after exposure to Y-27632.

In summary, our data demonstrate that Y-27632 inhibits the apoptosis of human keratinocytes expressing high levels of Myc. Synergy between Myc and Y-27632 results in long-term cell proliferation, which is accompanied by changes in the expression/modification of cell growth regulatory proteins. The most prominent alteration by Y-27632 is the generic inhibition of apoptotic pathways.

## MATERIALS AND METHODS

### Cell culture and immortalization

Primary human foreskin keratinocytes (HFKs) were isolated and cultured from neonatal foreskins as previously described [2, 9]. The cells were cultured in serum-free keratinocyte growth media (Invitrogen) supplemented with 50 μg/ml of bovine pituitary extract, 25 ng/ml of recombinant epidermal growth factor and gentamycin (50 μg/ml) (KGM media). Primary keratinocytes were transduced at passage 2 with amphotropic LXSN-based retroviruses containing empty vector with no insert (LXSN) or with Myc. The next day transduced cells were selected in 100 μg/ml G418 (Invitrogen, Carlsbad, CA) for about 5–7 days, or until control non-infected HFKs were completely dead. The LXSN and Myc cells were then cultured in synthetic KGM media with or without addition of 5 μM Y-27632 inhibitor and passed serially *in vitro* to assay for immortalization. Cells were passaged at a 1:8 ratio when 70–80% confluent. Therefore, each passage represents three cell population doublings. Cells were discarded if they did not reach 60% confluency after 3 weeks in culture. The cumulative number of days between passages was plotted against number of population doublings to obtain growth curves. All cells were cultured on plastic tissue culture dishes or flasks.

### Caspase-3 fluorometric assays

Keratinocytes were treated with the apoptotic inducers anti-human Fas (250 ng/ml) (MILLIPORE; Billerica, Massachusetts, USA; Cat # 05–201, clone: CH11), TNF-α (10 ng/ml) (Thermo scientific-Pierce; Rockford Illinois, USA; Cat # EN-RTNFAI), Etoposide (50 μM) (MP Biomedicals; Santa Ana, California, USA; Cat # ICN19391825), or Camptothecin (10 μM) (MP Biomedicals; Santa Ana California, USA; Cat # ICN15973280) with or without 24-h prior exposure to Y-27632. Cytosolic extracts were derived from pooled floating and attached cells and subjected to caspase-3 activity assays (Caspase-3 assay Kit; Life Technologies) using fluorescent tetrapeptide substrate specific for caspase-3 (Ac-DEVD-aminomethylcoumarin (AMC), BioMol, Plymouth Meeting, PA) as previously described [11]. Free AMC, generated as a result of cleavage of the aspartate-AMC bond, was monitored over 30 min with a Wallac Victor ^3^V fluorometer (Perkin Elmer, Waltham, MA) at excitation and emission wavelengths of 360 and 460 nm, respectively. The emission at 460nm from each sample was plotted against time, and linear regression analysis was used to determine the initial velocity (slope) for each curve, which yielded the activity. As a control, an inhibitor of caspase-3 (Z-DEVD-CHO; BioMol, Plymouth Meeting, PA) was added to cell extracts at a concentration of 50 μM 10 min prior to addition of the Ac-DEVD-AMC.

### RNA isolation and cDNA amplification

The cells were harvested and total RNA was purified with RNeasy Mini Kit (Qiagen, Germany) according to the manufacturer's instructions. Two μg of RNA were reverse transcribed with superscript III (Invitrogen, USA) according to the manufacturer's instructions.

### Quantitative real time PCR

The mRNA levels of target genes were determined using SYBR Green supermix (Bio-Rad) on a Bio-Rad iCycler and analyzed by an accompanying software (Bio-Rad Laboratories). All reactions were run in triplicates using approximately 50 ng/ml of cDNA obtained as described above.

The primer sets used were as follows: 5′-CTAGG AGATTTGTTTGGCGTGCC-3′ and 5′GCCGTTTTCG ACCCTGAGAG-3′ for p21; and 5′-TCTCCTCTGACTT CAACAGC-3′ and 5′GAAATGAGCTTGACAAAGTG-3′ for GAPDH. Taqman real-time QRT-PCR was performed on the Bio-Rad iCycler MyiQ for quantitation of hTERT mRNA using primers and probes (sense primer, 5′TGACACCTCACCTCACCCAC-3′, anti-sense primer, 5-CACTGTCTTCCGCAAGTTCAC-3′, and Taqman probe, 5′ACCCTGGTCCGAGGTGTCCCTGAG-3′) as previously reported [39, 49, 50]. The quantitative data were normalized by GAPDH and quantification of relative expression levels were estimated using the ΔΔCT method. Results were presented as relative fold change. Water was used as a negative control.

### Immunofluorescence staining

Cultured cells were grown on sterile glass cover slips and fixed with 3.7% paraformaldehyde at room temperature for 20 minutes. Cells were permeabilized by 0.1% saponin for 10 min and blocked with 10% donkey serum in PBS (Invitrogen, UK) for 20 minutes at room temperature. The cells were stained with the following primary antibodies: c-Myc Antibody (9E10) (Santa Cruz; 1:200), p53 (B-P3, Santa Cruz, 1:200), Caspase-3 Antibody (Cell Signaling; 1:400) or AlexaFluor 488 phalloidin (Life Technologies, 1:25). The corresponding secondary antibodies, AlexaFluor 488- and 555-conjugated donkey anti-mouse and anti-rabbit IgG (Invitrogen) were used at a concentration of 5 μg/mL, at room temperature for 1 hour. The cells were washed with PBS. Nucleic acid was stained with 0.5 μg/mL Hoechst stain (# 33342). The cover slips with cells were dropped onto a glass slide with mounting medium (Sigma-Aldrich, St. Louis, MO) and visualized with Zeiss Axioskop microscope (Carl Zeiss, Inc., Thornwood, NY). An ORCA-ER Digital Camera (Hamamatsu) was used for visualization and microphotography. The caspase-3 activity in detached cells was measured using using Image-iT Live green caspase detection kit (Life Technologies), as recommended by the manufacturer.

### Western blot analysis

Cell lysates were prepared using 2X SDS gel electrophoresis sample buffer. Proteins were separated by 4–20% gradient Tris-glycine gel (Invitrogen, Carlsbad, CA) electrophoresis, and Immobilon-P PVDF membrane (Millipore) transfer was performed as previously described [2, 9]. Membranes were blocked in 5% dry milk-PBST (0.1% Tween 20 in PBS) and probed with primary antibodies at dilution of 1:1000. The primary antibodies used were as follows: pRB antibody (Cell Signaling), P-pRB (Cell Signaling), p53 (B-P3, Santa Cruz), p-p53 S15 (Cell Signaling), p16 (N-20, Santa Cruz), Myosin Light Chain 2 (MLC2; Cell Signaling), P-MLC2 (Thr18/Ser19; Cell Signaling), or P-cofilin (Cell Signaling). The membranes were then probed with anti-rabbit IgG or anti-mouse IgG secondary antibodies conjugated to HRP (Santa Cruz Biotechnology). The membranes were visualized by using Western Blotting Chemiluminescence Luminol Reagent (Santa Cruz). As a loading control, membranes were probed with an antibody specific for β-actin (Sigma-Aldrich, St. Louis, MO) at 1:10,000. Membranes were stripped using Restore Western Blot Stripping Buffer (Pierce, Rockford, IL).

### Microarray assays

The Illumina HumanHT-12 v4 Gene Expression Beadchip Kit microarray platform was used to process the samples and obtain the raw data. The raw data was first imported into Illumina Genome Studio to be pre-processed, and the result was analyzed by Partek Genomics Suite Software. Mixed model analysis of variance was performed, and multiple comparisons were made based on the experimental design. For each comparison, a list of significant genes was gained by the cut-off of absolute fold change greater than 2. Intersections were made among multiple significant gene lists, and the corresponding Venn diagrams were obtained. For selected interested intersections of significant gene lists, Qiagen Ingenuity Pathway Analysis software was used to discover the corresponding pathways and/or gene networks. Fisher's exact tests were used to test all canonical pathways and Gene Ontology Biological Functions, to check whether or not they are enriched on particular significant gene lists or the intersections of them, and a list of significant pathways and biological functions were reported based on the cutoff of Benjamini-Hochberg false discovery rate adjusted *p*-value < 0.05.

### Statistical analysis

Results were presented as mean ± standard deviation. Statistical significance was defined as *p* < 0.05, using two-tailed Student *T*-tests. All experiments were repeated at least two times in triplicate and results of representative experiments are shown.

### Ethics statement

The HFK cells were prepared from human neonatal foreskins at Georgetown University Hospital. Normally these tissues are de-identified and discarded. The protocol (2002–021) has been approved by the Georgetown University Institutional Review Board.

## SUPPLEMENTARY MATERIAL FIGURES


