# Supplementary material for: ROCK inhibitor reduces Myc-induced apoptosis and mediates immortalization of human keratinocytes
Source: Oncotarget. 2016 Aug 20;7(41):66740–53. doi: 10.18632/oncotarget.11458 (PMC5341834; doi:10.18632/oncotarget.11458)
Supplement: Supplementary file 1 [file oncotarget-07-66740-s001.pdf]

ROCK inhibitor reduces Myc-induced apoptosis and mediates immortalization of human keratinocytes

Supplementary Materials

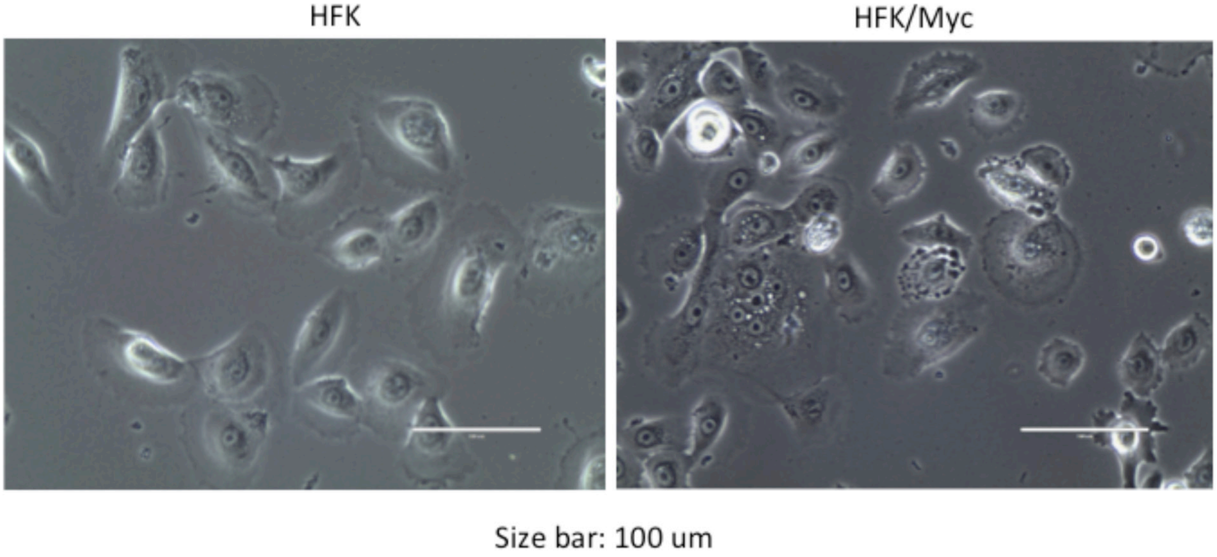

Supplementary Figure S1: Cell Morphology of control or Myc-expressing keratinocytes.

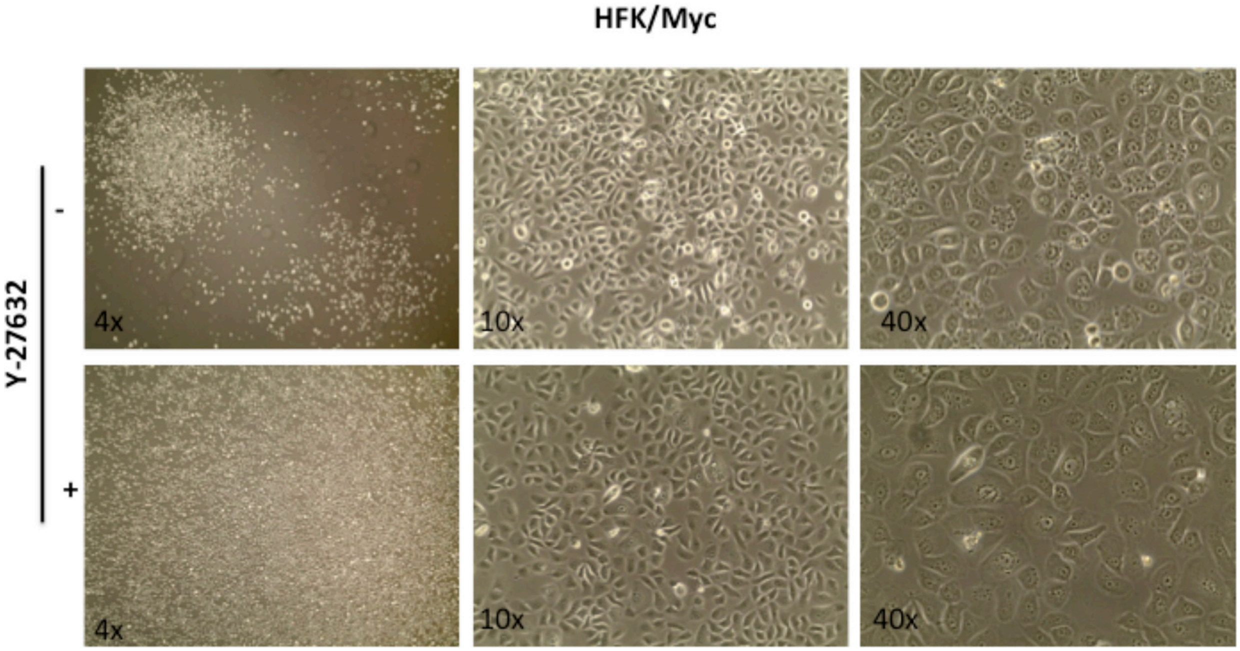

Supplementary Figure S2: Cell Morphology of Myc-expressing keratinocytes in presence or absence of Y-27632.

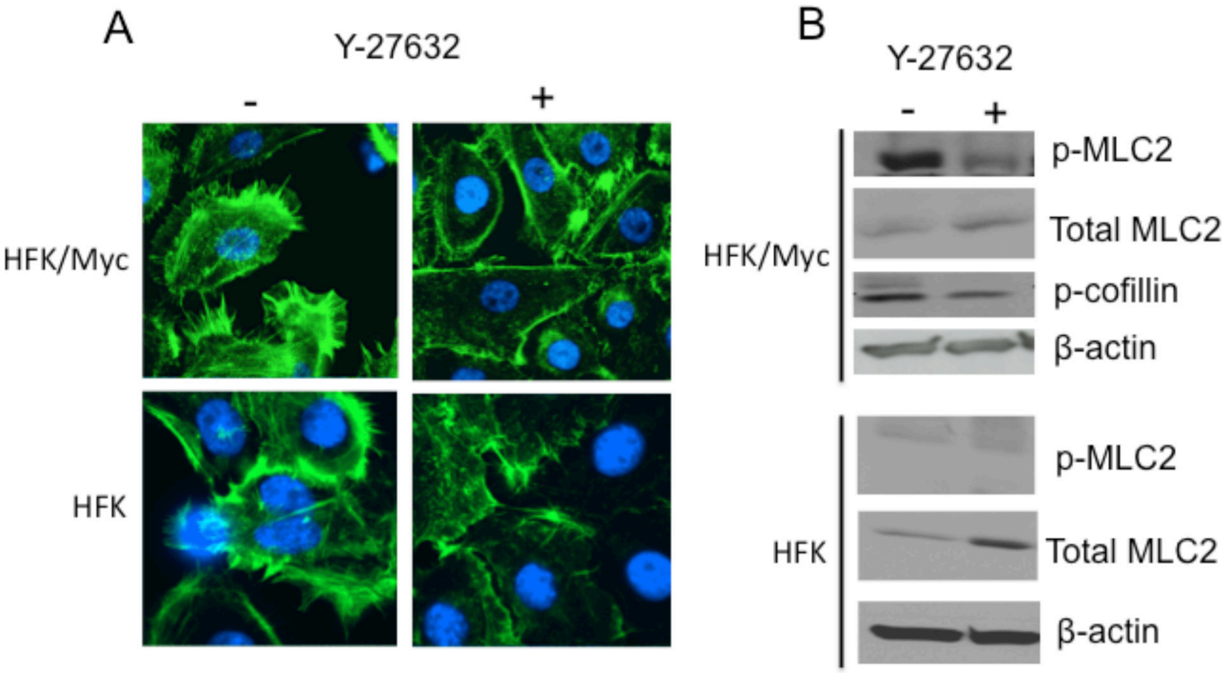

Supplementary Figure S3: Rock inhibitor (Y-27632) alters the actin cytoskeleton. (**A**). Y-27632 reduces actin stress fibers in Myc-expressing or control keratinocytes. HFK/Myc cells were treated with 5 μM Y-27632. Cells were stained with rhodamineconjugated phalloidin, which specifically binds to polymerized actin microfilaments (Factin). Disruption of total ROCK activity by inhibition with Y-2763, results in a dramatic reorganization of F-actin in both control and Myc-expressing keratinocytes. (**B**). Y-27632 decreases the phosphorylation of myosin light chain2 (MLC2) and cofilin. The cell extracts were subjected to immunoblot analysis using antibodies to P-cofilin, MLC2, PMLC2 and β-actin as a loading control.

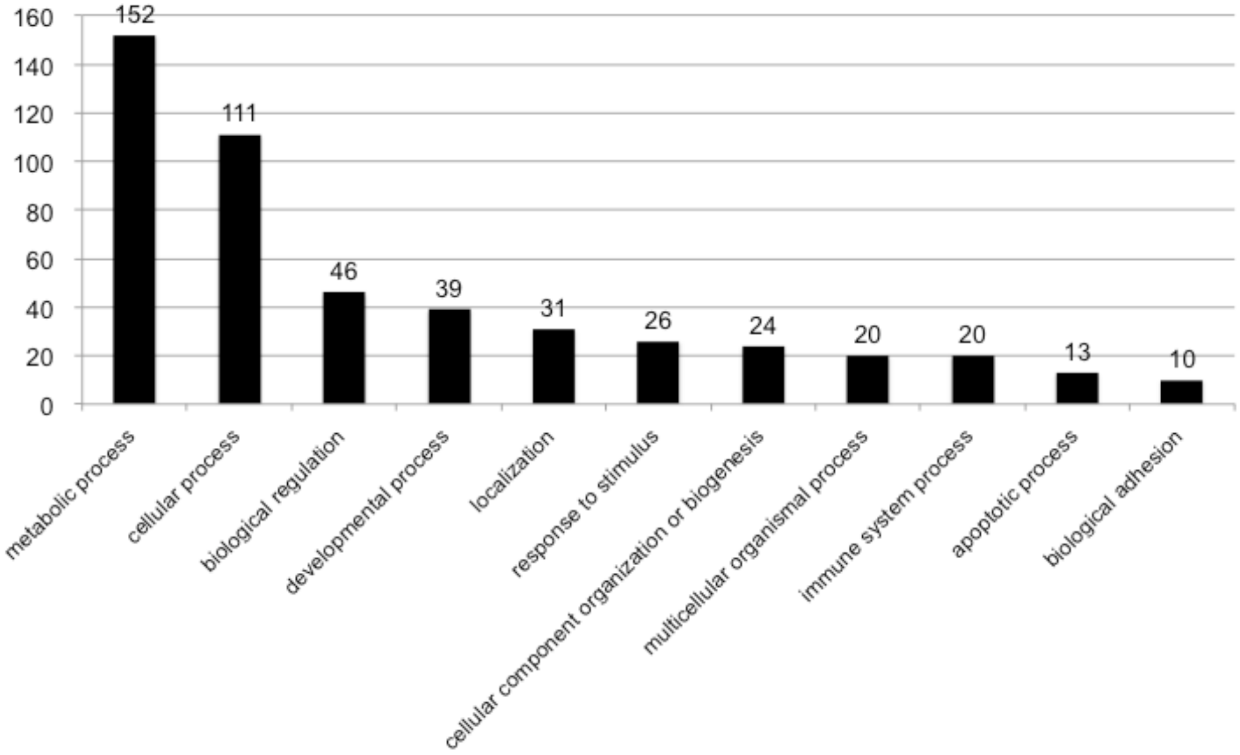

Supplementary Figure S4: Y-27632 alters the profile of gene expression associated with apoptosis. Y-27632 reduced Myc-induced apoptosis of human keratinocytes and immortalized Myc-expressing keratinocytes in serum free synthetic medium. Microarray assays were performed and the categories of genes were analyzed using DAVID software. Y-27632 altered gene expression involved in metabolic processes, cellular processes, biological regulation, response to stimuli, cellular component organization or biogenesis, and apoptotic process, *etc*.

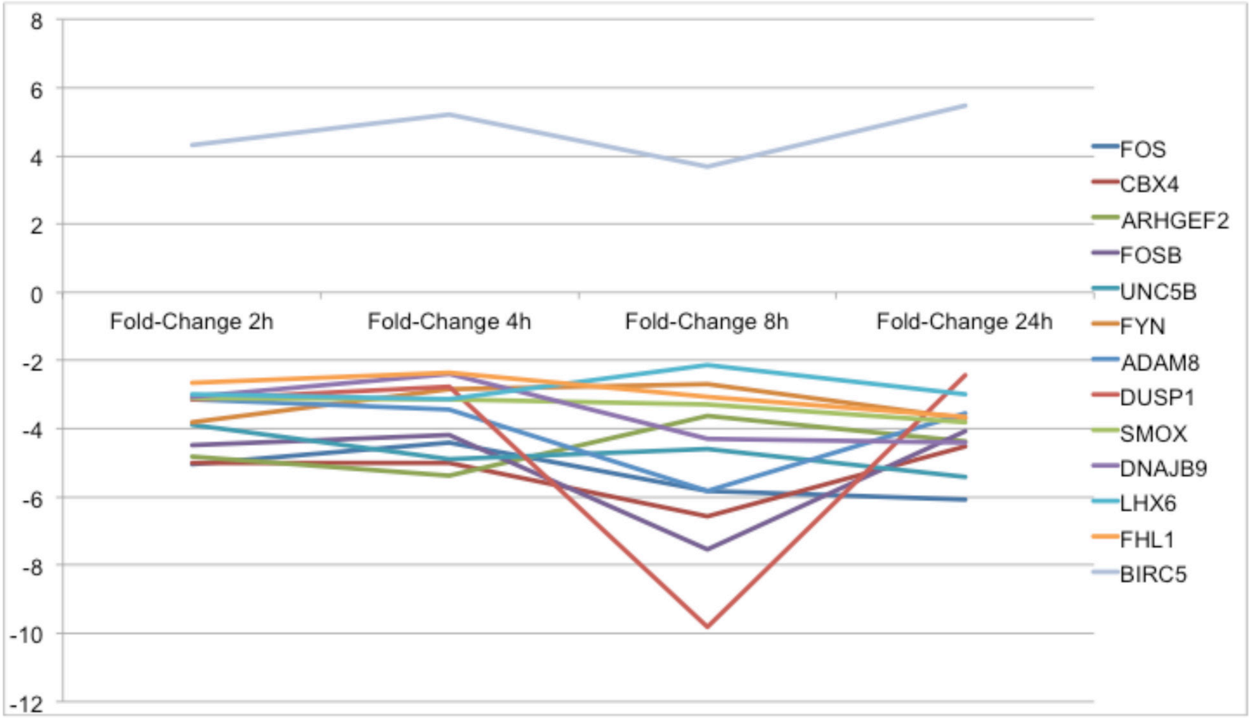

Supplementary Figure S5: Survivin was the only one apoptosis-related gene which was upregulated at 2, 4, 8 and 24 hrs, while the other 12 genes were downregulated.
